# Systematic Review of *IL-1*, *IL-4*, *IL-6*, *IL-10*, *IL-15*, and *IL-18* Gene Polymorphisms and Meta-Analysis of *IL-6* Variant and Its Association with Overweight and Obesity Risk in Men

**DOI:** 10.3390/ijms252413501

**Published:** 2024-12-17

**Authors:** Aleksandra Bojarczuk, Aleksandra Garbacz, Cezary Żekanowski, Beata Borzemska, Paweł Cięszczyk, Ewelina Maculewicz

**Affiliations:** 1 Faculty of Physical Culture, Gdansk University of Physical Education and Sport, 80-336 Gdansk, Poland; c.zekanowski@imdik.pan.pl (C.Ż.); beatab@imdik.pan.pl (B.B.); pawel.cieszczyk@awf.gda.pl (P.C.); 2 Faculty of Animal Genetics and Conservation, Warsaw University of Life Sciences, 02-787 Warsaw, Poland; aleksandra_garbacz1@sggw.edu.pl; 3 Department of Neurogenetics and Functional Genomics, Mossakowski Medical Research Institute, Polish Academy of Sciences, 02-106 Warsaw, Poland; 4 Faculty of Physical Education, Jozef Pilsudski University of Physical Education in Warsaw, 00-968 Warsaw, Poland; ewelina.maculewicz@awf.edu.pl; 5 Department of Laboratory Diagnostics, Military Institute of Aviation Medicine, 01-755 Warsaw, Poland

**Keywords:** obesity, overweight, interleukin, genetic variant, polymorphism

## Abstract

Obesity is a complex health risk influenced by genetic, environmental, and lifestyle factors. This review systematically assessed the association between interleukin gene polymorphisms (rs16944, rs17561, rs1143623, rs1143633, rs1143634, rs1800587, rs2234677, and rs4848306), *IL-4* (rs180275, rs1805010, *IL-6* rs13306435, rs1800795, rs1800796, rs1800797, rs2228145, rs2228145, rs2229238, and rs4845623), *IL-10* (rs1518110, rs1518111, rs1800871, rs1800872, rs1800896, rs1878672, rs2834167, rs3024491, rs3024496, rs3024498, and rs3024505), *IL-15* (rs3136617, rs3136618, and rs2296135), and *IL-18* (rs187238, rs1946518, rs2272127, rs2293225, and rs7559479) and the risk of overweight and obesity in adults, focusing on *IL-6* rs1800795 through a meta-analysis. The focus on *IL-6* in this review arises from its pleiotropic nature and unclear effect on obesity risk. The review included studies published from 1998 to 2023, sourced from Science Direct, EBSCOhost, Web of Science, and Google Scholar. Bias was assessed with the Cochrane Collaboration tool, and funnel plots were used for publication bias. Results were synthesized into pooled odds ratios (ORs) and confidence intervals (CIs). Thirty studies comprising approximately 29,998 participants were included. The selection criteria required that the articles include participants who were overweight or obese, and this condition needed to be linked to *IL* polymorphisms. In a meta-analysis, in the dominant model, the pooled OR was 1.26 (95% CI 1.08 to 1.47), indicating those with the GC/CC genotype for *IL-6* rs1800795 are 1.26 times more likely to be overweight/obese than GG genotype carriers. For the recessive model, the OR was 1.25 (95% CI 1.04 to 1.51). The overdominant model showed no significant association (OR 1.08, 95% CI 0.94 to 1.25). Interleukin gene variation, particularly the *IL-6* rs1800795 variant, is modestly associated with obesity risk. This suggests that other factors, such as the environment, also play a role in obesity. Thus, individuals with this particular *IL-6* variant may have a slightly higher likelihood of being overweight or obese compared to those without it, but this is just one of many factors influencing obesity risk.

## 1. Introduction

Obesity is a significant health risk in today’s society, accounting for an increasing proportion of the global burden of non-communicable diseases, such as diabetes, cardiovascular disease, hypertension, and some cancers [1]. Over the past four decades, obesity rates have tripled, with recent reports estimating that nearly 2 billion adults (39% of the global adult population) are overweight and 671 million (12% of adults worldwide) are now obese [2]. Current trends project that nearly 20% of the world’s adult population will be obese by 2025 [2].

Genetic factors play a significant role in an individual’s response to the obesogenic environment, contributing to the global rise of obesity. Hereditary factors account for 40% to 50% of the variability in human body weight. However, this factor is less critical in normal-weight individuals (about 30%) and significantly higher in the subgroup of individuals who are considered obese, particularly in those with severe or morbid obesity (about 60–80%) [3]. Some evidence from previous studies suggests that interleukin system polymorphism may be related to obesity [4,5,6]. However, sex, age, geographic differences, and population-specific or ethnic group-specific effects may influence the associations between gene variants and obesity. Genome-wide association studies (GWAS) for body mass index (BMI), waist-to-hip ratio, and other adiposity traits have identified more than 300 single-nucleotide polymorphisms (SNPs; all SNPs are designated by numbers beginning with “rs”). There has been limited discussion on cytokine genes and overweight/obesity despite their implication for this condition [7]. Cytokine genes have not received much attention, even though they are critical in inflammation and controlling adipose tissue metabolism [8,9]. The effects of cytokine genes on overweight and obesity across different models support the hypothesis that variations in interleukin genes play a role in these conditions. For instance, overexpressing *IL-4* transgenic mice demonstrated a significant loss of dermal adipose tissue, suggesting that IL-4 suppresses adipocyte formation and lipid storage [10]. Similarly, mice lacking IL-18 gained 2–3 times more body weight per unit of energy consumed on a low- or high-fat diet, indicating the protective effect of IL-18 against obesity [11]. The blocking of IL-15 [12] and IL-6 [13] in humans and mice is associated with obesity. A recent meta-analysis indicated that the minor alleles of rs1800795 in the *IL-6* gene may be linked to an increased risk of obesity [4]. However, two studies conducted in Poland did not reveal a clear association between rs1800795 and obesity [14,15].

This review delves into the genetic factors related to overweight and obesity, explicitly highlighting interleukin gene polymorphisms. The rationale for this focus is the crucial role of interleukins in inflammation and metabolic regulation, which are significant in overweight and obesity. Therefore, this review systematically examines the association between interleukin gene polymorphisms (specifically *IL-1*, *-4*, *-6*, *-10*, *-15*, and *-18*) and the prevalence of overweight and obesity. The review aims to analyze existing studies from 1998 to 2023 to identify potential genetic determinants that contribute to overweight and obesity risk, with a particular focus on the *IL-6* rs1800795 variant. The timeline of significant milestones in obesity genetics began in 1997 with the initiation of candidate gene studies. This was followed in 1998 by the first genome-wide linkage studies, marking a pivotal moment in our understanding of the genetic factors contributing to obesity. A further breakthrough occurred in 2005 with the introduction of genome-wide association studies [16]. Given these developments, focusing on the period starting in 1998 would provide the most relevant and comprehensive overview of the literature in this field. The manuscript was completed in early 2024, and due to the review process, the final submission was considered in late 2024. Given the scope of the review, studies up to 2023 were deemed sufficient for addressing the research questions, ensuring the analysis reflects the most current understanding of the topic. A recent meta-analysis suggested that the minor alleles of rs1800795 in the *IL-6* gene could be associated with a higher risk of obesity [4]. Nevertheless, two studies in Poland did not show a definitive connection between rs1800795 and obesity [14,15]. This suggests that the genetic variation at this specific polymorphic site could be significant in understanding the underlying genetic factors contributing to obesity. Therefore, to contribute to the ongoing discussion regarding the genetic predictors of obesity and the inconsistencies observed in different populations, we decided to evaluate *IL-6* rs1800795.

## 2. Materials and Methods

### 2.1. Design

This study is a systematic review of the relationship between genetic variations in interleukin genes and obesity and a meta-analysis of the association between *IL-6* rs1800795 and obesity. We followed the Preferred Reporting Items for Systematic Reviews and Meta-Analysis (PRISMA) guidelines [17].

### 2.2. Eligibility Criteria

We selected research articles published between 1998 and 2023 to yield results that reflected genetic studies on the association of *IL* polymorphisms with overweight/obesity. Full works were assessed for eligibility according to the inclusion and exclusion criteria. The exclusion criteria encompassed non-human studies and reviews. The specifics of a research group were not a determining factor. The studies sought pertained solely to specific rs and constituted research articles, as opposed to reviews or general descriptions of the impact of entire proteins/genes. Studies with children, pregnant or lactating women, severe diseases, and surgical patients were also included.

### 2.3. Search Strategies

Four scientific databases (Science Direct, EBSCO host, Web of Science, and Google Scholar) were searched using the following keywords: interleukin, genes, obesity, overweight, and physical activity. Further searches were performed using the following terms: medicine and dentistry, biochemistry, genetics, and molecular biology, available articles only (Science Direct), obesity, body mass index, biomarkers, descriptive statistics, and language English (EBSCO host). The decision to exclude Scopus from the search was informed by including other established databases, such as PubMed, Web of Science, and Google Scholar, which collectively provide extensive coverage of the relevant literature within this domain. While Scopus is recognized as a significant resource, the databases chosen were considered adequate for conducting a comprehensive and rigorous search of high-quality, peer-reviewed studies. The value of Scopus has been acknowledged, and its consideration for future searches will be deemed appropriate.

### 2.4. Study Selection and Data Extraction

**Figure 1:**
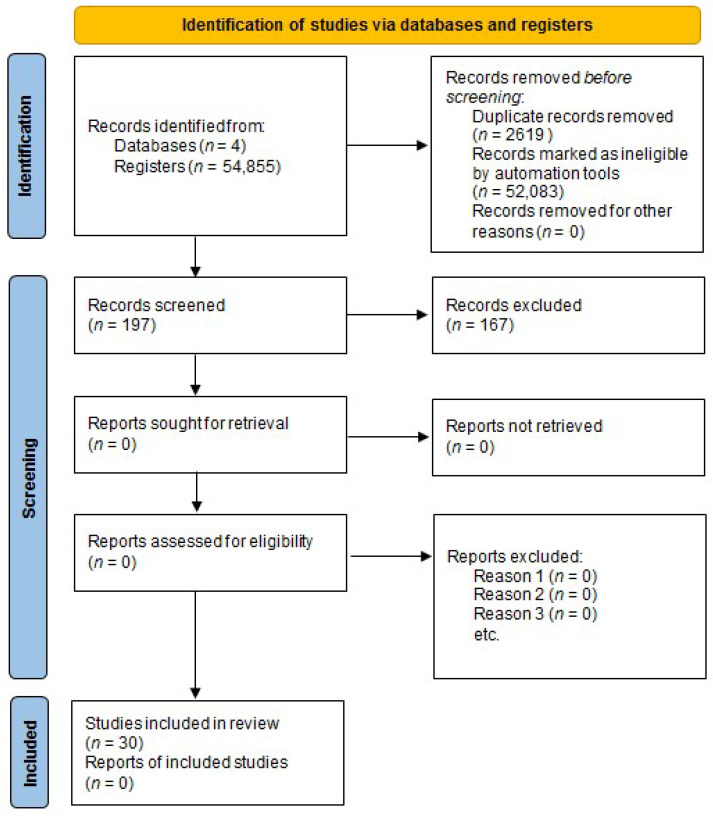
PRISMA flow chart for the study selection. Adapted from [17].

Data were extracted using the Systematic Review Accelerator (SRA) software (https://sr-accelerator.com/#/ (accessed on 1 October 2023)), which ensured accuracy and managed redundant studies through its Deduplicator and Screenatron functions. The collected data were shared with co-authors using Excel version 14.0.7268.5000 (32-bit) and EndNote 20.0. Initially, one author screened all studies by title to exclude irrelevant ones, followed by independent abstract reviews by two authors. For potentially eligible studies, full texts were assessed by two authors against inclusion criteria. Disagreements were resolved by consensus or, if necessary, by a third author. Extracted data included gene, polymorphism, cohort size, population (ethnic groups), and participant characteristics related to overweight/obesity. A single author gathered and saved data on a portable drive and in the cloud, allowing two additional authors to access it and work independently. Additionally, the data were stored in EndNote 20.0 and Excel, which all co-authors had access to (Figure 1).

### 2.5. Risk-of-Bias Assessment

The Cochrane Collaboration tool [18] was utilized for the review to evaluate bias risk. For the meta-analysis, funnel plots were utilized. To ensure the accuracy of the evaluation process, any identified discrepancies were resolved through discussion. Additionally, a funnel plot was employed to assess the potential for publication bias and minimize any potential biases in the analysis.

### 2.6. Data Synthesis and Meta-Analysis

For the systematic review, tables were utilized to categorize article contents for descriptive analyses. In conducting the meta-analysis, R-4.0.3 was employed. Among the 10 articles, the influence of genotype on BMI was primarily examined, with 6 articles excluded based on the funnel plot and the Baujat method. These articles encompassed results for males. Meta-analysis utilized fixed-effect and random-effect models, with a 95% confidence interval, pooled odds ratio, and weight assigned to each article in every meta-analysis. The heterogeneity among the articles was assessed using the I^2^ index.

### 2.7. Statistical Analysis

All statistical analyses were performed using R software (version 4.0.3, R Foundation for Statistics Computing, https://cran.r-project.org/ (accessed on 1 October 2023)). The Mantel–Haenszel method was used to calculate the overall OR from all the data in one analysis. The Woolf test was applied to test for the heterogeneity of studies, enabling a meta-analysis. These calculations were made using the meta function from the meta.bin package in R (version 4.0.3.) Sensitivity analysis, detecting different studies affecting the lack of homogeneity of groups, was performed using the Baujat graph, created using the Baujat function, and the funnel plot, created using the funnel function in the R meta package. A forest plot was chosen to summarize the collective analysis of the influence of the *IL-6* variant (rs1800795) on BMI because it provides a graphical representation of each study’s observed effect, CIs, and weight. The Chi-squared test and I^2^ statistic were used to determine statistical heterogeneity. The *p*-value of the chi-squared test represents the probability of the null hypothesis that there is no heterogeneity between studies. If the *p*-value was less than 0.05, we considered that there was heterogeneity between the studies [19]. Additionally, the I^2^ statistic was calculated to determine the percentage of the observed variance resulting from the actual difference in the magnitude of the effects studied and to answer the question of how much heterogeneity is present.

A random-effect model was selected for our analyses due to high heterogeneity (I^2^ > 40% and *p*-value < 0.05). This model calculates the cumulative effect based on the weighted average of the effects of the individual studies. The weights are determined as the inverse of the variance within the studies, increased by the variance between the studies. A fixed-effect model was chosen when I^2^ < 40% and *p*-value > 0.10. In this model, it is assumed that the results of all studies describe the same actual effect size and that the differences in the observed effects are due to sampling error. In this case, the cumulative effect is determined based on the weighted average of the impact, where the weights are defined as the inverse of the variance of each study.

The OR was chosen to measure the effect of interest because this coefficient was used in all articles reviewed here. Specifically, we calculated an overall OR representing the weighted averages of the individual study estimates.

## 3. Results and Discussion

### 3.1. Study Selection

The literature search revealed 54855 publications. After removing 2619 duplicates and 52,039 records marked as ineligible by automation tools, 197 were screened based on the title, of which 61 were excluded. Next, 136 full-text articles regarding interleukins were read for detailed evaluation, of which 33 were rejected after reading as incompatible with the topic. The following were retained for description and further analysis: *IL-1* (*n* = 20), IL-4 (*n* = 2), *IL-5* (*n* = 1), IL-6 (*n* = 55), *IL-8* (*n* = 3), *IL-10* (*n* = 11), *IL-15* (*n* = 2), *IL-16* (*n* = 1), *IL-17* (*n* = 2), and *IL-18* (*n* = 6). The final number of articles included was 30 (Figure 1). The selection process was performed manually. A consistent research methodology across all articles is crucial for the meta-analysis of individual variants. Only *IL-1* rs1143634 (*n* = 3), *IL-6* rs1800795 (*n* = 4), and *I-L6* rs1800796 (*n* = 2) met the criteria. However, due to insufficient samples, *IL-1* rs1143634 and *IL6* rs1800796 were excluded from further analyses.

### 3.2. Study Characteristics

Table 1 and Table 2 outline the characteristics of the studies (population and cohort; age, body composition, and additional information). The types of publications were research articles, not reviews. Of the 30 articles, 13 were case–control studies [6,20,21,22,23,24,25,26,27,28,29,30,31], 1 of longitudinal design [32], and 16 were cross-sectional associations [5,33,34,35,36,37,38,39,40,41,42,43,44,45,46,47]. The participants were exclusively male (nine publications [22,23,32,38,40,41,43,45,46]), exclusively female (three publications [5,20,29], females and their newborns of unknown sex [34], and sometimes included both sexes (17 publications [6,21,24,25,26,27,28,30,31,33,35,36,37,38,39,44,47]).

### 3.3. Risk of Bias

**Figure 2:**
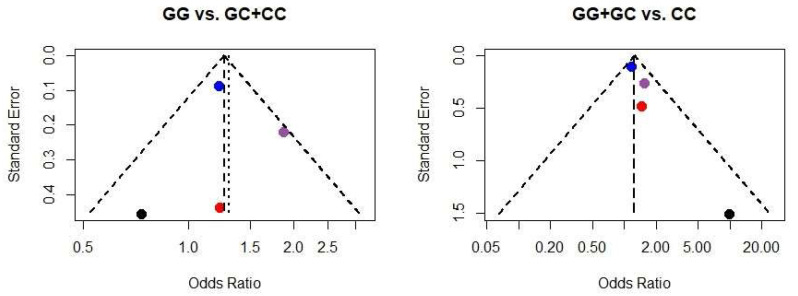

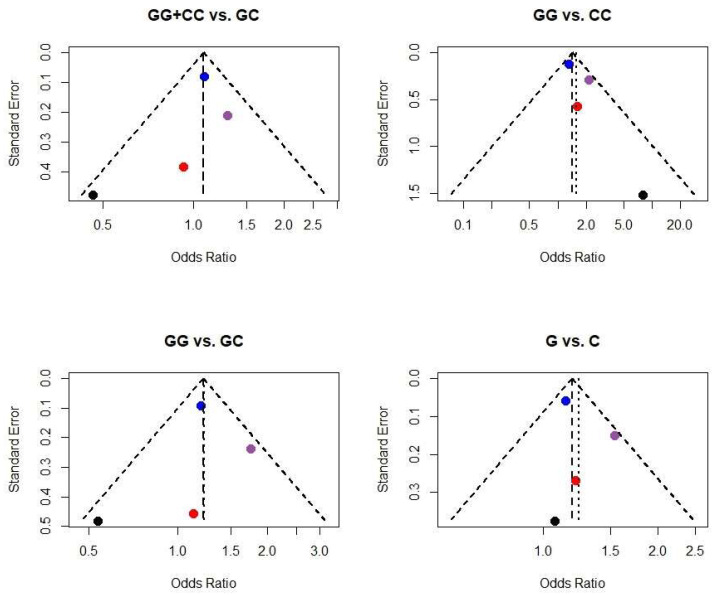
Funnel plots displaying the relationship between the effect size of individual studies (x-axis) and their precision, expressed as the inverse of the standard error (y-axis). Each dot represents a single study included in the meta-analysis. The blue dot represents Stephens et al., 2004 [22], purple Wernstedt et al., 2004 [33], red Maculewicz et al., 2021 [23], and black Cimponeriu et al., 2013 [27].

**Figure 3:**
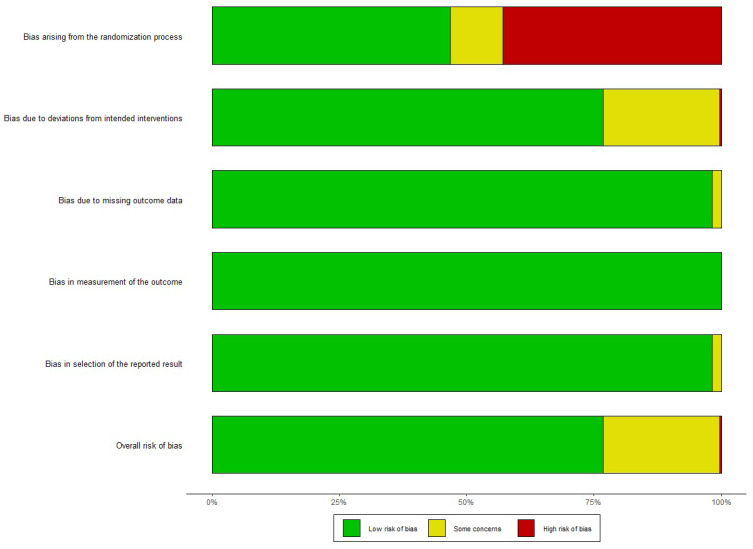
Risk assessment for the review applying the Cochrane Collaboration tool [17].

For our meta-analysis, a funnel plot was used to assess the possibility of publication bias (Figure 2). The plot is symmetric when publication bias is minimal [48]. The review used the Cochrane Collaboration tool [18] to assess the risk of bias (Figure 3). Using a small number of articles in our study was considered a risk factor because it could affect the accuracy of the results. A quality assessment graphic was created to visually summarize the evaluation of the included studies (Table 3).

### 3.4. Review

#### 3.4.1. IL Gene Polymorphisms and Overweight/Obesity

**Figure 4:**
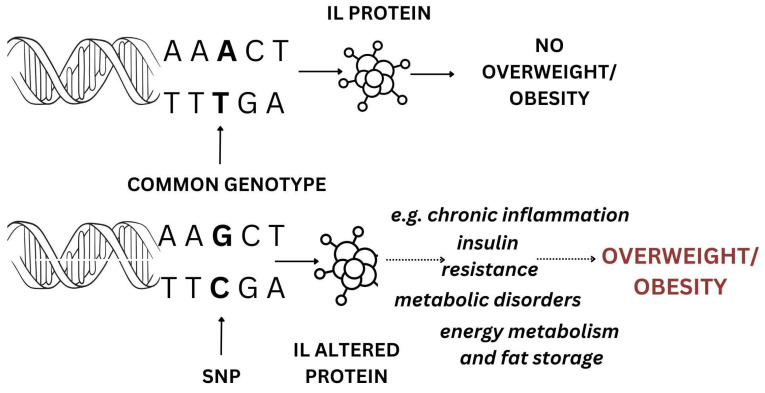
A simplified mechanism for the association between interleukin gene polymorphisms and overweight/obesity.

Obesity can be influenced by genetic factors, which fall into two primary categories. The first category is called monogenic obesity, characterized by a Mendelian inheritance pattern. This form is relatively rare, typically emerges in early childhood, usually exhibits severe symptoms, and is linked to particular chromosomal deletions or mutations in single genes. The second category, known as polygenic obesity or common obesity, results from the combined effects of various genetic polymorphisms, each exerting a small effect on an individual’s predisposition to obesity [16]. Interleukins are a group of cytokines that play key roles in immune responses, inflammation, and metabolic processes. As Section 3.4.2, Section 3.4.3, Section 3.4.4, Section 3.4.5, Section 3.4.6 and Section 3.4.7 describe, polymorphisms in *IL* genes may influence the risk of overweight and obesity. Various studies mentioned indicate associations between specific variants and fat mass or BMI in different populations (see Section 3.4.2, Section 3.4.3, Section 3.4.4, Section 3.4.5, Section 3.4.6 and Section 3.4.7). Thus, the association between *IL* gene polymorphisms and overweight/obesity can be elucidated through various biological mechanisms. The possible simplified biological mechanism for the association between the interleukin gene polymorphisms and overweight/obesity is demonstrated in Figure 4. Genetic polymorphisms in these genes can influence cytokine production, which may subsequently affect pathways related to energy balance, fat storage, and metabolic regulation.

#### 3.4.2. IL-1 Gene Complex Polymorphisms

Strandberg et al. (2006) found that the *IL-1B* rs1143634 (+3953 C/T) common polymorphism was associated with fat mass in 1068 young men. The study showed that carriers of the rs1143634 T variant (CT/TT) had a significantly lower total fat mass and reduced arm, leg, and trunk fat than rs1143634 CC subjects [40]. However, a further investigation found no association with total fat mass in 3014 older men (69–81) [41]. Another study confirmed a significant decrease in the frequency of the rs1143634 T variant in the overweight group in 181 healthy females with a marked variation in body mass index [5]. A study by Manica-Cattani et al. (2010) conducted on 880 Caucasian females and males (aged 59.7 ± 11.9 years) reported that rs1143634 CC carriers had 1.340 (95% CI: 1.119–1.605) and 1.623 (95% CI: 1.349–1.95) times more chance of being obese and overweight, respectively. A higher rs1143634 T variant (CT/TT) frequency in the non-overweight group was confirmed. Regression analysis indicated that the observed association was independent of sex (Wald 0.145, *p* = 0.704) and age (Wald 0.156, *p* = 0.693) [42]. Furthermore, an association was observed between total and regional fat mass and *IL-1B* rs1143627 (−31 T/C), but not with lean body mass, as reported by Strandberg et al. (2008) [41]. A recent study of 292 men (mean age 46.5 years) revealed significant interactions between IL-1 genetic variation, central adiposity, and thus inflammation-induced periodontitis progression. However, the baseline values of body mass index (BMI), waist circumference, or waist-to-height ratio were not associated with *IL-1* genotype, i.e., *IL-1A* rs17561 (+4845 G/T), *IL-1B* (rs1143634, rs1143623 (−1464 G/C), rs4848306 (−3737 C/T), rs1143633 (+3877 G/A), and rs16944 (−511 C/T)) [32]. This is consistent with the study by Maculewicz et al. (2022), where the *IL-1B* rs1143634, *IL-1A* rs1800587 (−889 C/T), and *IL-1RN* rs2234677 (−87 G/A) variants were not associated with BMI nor fat percentage in the tested group of 101 physically active male cadets (aged 19–25 years). Despite the lack of association of individual variants and genotypes with obesity, it has been shown that the co-occurrence of *IL-1B* rs1143634 TC x *IL-1RN* rs2234677 GG genotypes could be a protective factor against overweight and obesity in Caucasians [43].

#### 3.4.3. IL-4 Gene Polymorphisms

In contrast to IL-1, less evidence has been found for obesity-related SNPs in the *IL-4* gene. Ha et al. (2008) showed that *IL-4R* rs180275 (+1902 A/G; Q576R) was associated with BMI in 876 Koreans. The frequency of the rs180275 G variant is significantly lower in subjects with very high BMI, suggesting a protective role against obesity in the Korean population. A similar association was not found for another non-synonymous rs1805010 (+4679 A/G; I75 V) variant [44].

#### 3.4.4. IL-6 Gene Polymorphisms

Berthier et al. (2003) were the first to report the association of the *IL-6* rs1800795 polymorphism with obesity in 270 healthy French-Canadian men [38]. The G variant was more frequent in lean individuals (associated with BMI < 25 kg/m^2^ and waist circumference < 100 cm), while carriers of the C variant had a larger waist. The authors concluded that rs1800795 may predict adiposity in men [38]. This is in line with the study by Wernstedt et al. (2004), who examined 74 healthy women (aged 38 years) and 485 hypertensive subjects (aged 57 years, 73% male). They found that C-containing genotypes of the *IL-6* rs1800795 were associated with increased BMI [33]. Similarly, the rs1800795 CC genotype was more common in obese individuals in a case–control study of 150 obese and 150 normal-weight Caucasian subjects (110 men and 40 women in each group [27]). Moreover, an association between the *IL-6* rs1800795 C variant and BMI was confirmed in 571 Caucasian type 2 diabetics of both sexes (mean age 56.1 ± 3.5 years old) [22] and in 668 men and women (aged 40 to 64 years) participating in the studies investigating the association between diet and cancer and other chronic diseases [25]. The study involving 370 unrelated North Indian women (192 with abdominal obesity and 178 controls) also confirmed a significantly higher prevalence of the C-containing genotype in obese women [20]. Notably, a higher BMI and waist circumference trend was observed in the healthy Caucasian carriers of the rs1800795 C variant (52 women and 54 men aged 20–40). Additionally, the authors concluded that carrying the rs1800795 C variant increases the likelihood of developing hypertension and insulin resistance, which are reliable indicators of future cardiovascular disease [24]. The study by Panoulas et al. (2008) confirms a significant prevalence of cardiovascular disease in overweight/obese rheumatoid arthritis (RA) patients carrying the *IL-6* rs1800795 C variant. However, no significant interactions between adiposity and rs1800795 were observed in 383 RA patients (61.3 ± 12.1 years) or 422 non-RA patients (50.1 ± 15.7 years) [35]. The study by Boeta-Lopez et al. (2018) in a Mexican-American cohort of 146 men and 291 women (aged 17 ± 8.26 years) showed a higher frequency of the A variant in *IL-6* rs1800797 (−597 G/A) among individuals without obesity, suggesting a possible protective effect, despite the lack of statistical significance. This study also demonstrated a significant prevalence of the C variant in *IL-6* rs1800796 (−572 G/C) among subjects with increased waist circumference [36]. Another study showed that the rs1800796 C variant was associated with obesity in 222 Caucasian children [21]. In contrast, the G variant protected against gestational weight gain in 309 Romanian mothers [34]. This observation contrasts the study above (Wernsted et al., 2004) in healthy and hypertensive subjects [33]. *IL-6* promoter polymorphisms rs1800795, rs1800796, and rs1800797 also did not influence obesity in a case–control study of multiple myeloma and plasmacytoma, including 150 cases and 126 controls (similar gender distribution, aged 24 to 74 years) [26]. Likewise, the study of 125 Polish, physically active male military personnel (aged 19–26 years) did not confirm the association between BMI and percent body fat and *IL-6* rs1800795, rs1800796, and rs13306435 polymorphisms [23]. What is more, the genotype–quantitative trait interaction study conducted in 4401 middle-aged (aged 30–45 years), glucose-tolerant Caucasian Danes suggested that rs1800795, but not rs1800796, could explain the inter-individual heterogeneity in BMI. In contrast to the previously mentioned studies, carriers of the rs1800795 G variant (GG/GC) had a statistically higher BMI than carriers of the CC genotype. Furthermore, the lean phenotype was associated with a higher frequency of the AGC haplotype (rs1800797-rs1800796-rs1800795) when compared to the obese phenotype with the prevalence of the GGG haplotype [28].

A study conducted on approximately 700 non-diabetic Pima Indians (aged 36 ± 11–39 ± 12 years), a population prone to excess adiposity, highlighted the importance of genetic variability in the IL-6 receptor (*IL-6R*). Five reported variants in strong linkage disequilibrium, rs4845623, rs2228145, rs2229328, c_1981308_10, and c_1158918_10, were associated with obesity. Individuals carrying the rarer variants had a higher mean BMI than those with the wild-type variants. However, the statistical significance of the association varied as a function of allele frequency [39].

#### 3.4.5. IL-10 Gene Polymorphisms

Obese patients have elevated IL-10 levels [49,50]. Scarpelli et al. (2006) studied the *IL-10* rs1800896 (−1082 G/A), rs1800871 (−819 C/T), and rs1800872 (−592 C/A) polymorphisms in 551 type 2 diabetic and 1131 control Italian Caucasians. Although rs1800872 was not associated with diabetes, non-diabetic homozygous A carriers had increased BMI and insulin resistance compared with the other genotypes [6].

In a homogenous cohort of 131 cadets of the Military University (aged 22.4 ± 2.2 years), the CCGTA haplotype (*IL-10* rs1518111, rs1878672, rs3024496, rs3024498, and rs3024505) was found to be more than twice as frequent in the control group than in the group with a high BMI. The researchers also reported an association between the *IL-10* rs3024505 variant and obesity. The risk of obesity assessed by high body fat percentage was 4.7 times lower in rs3024505 AG carriers compared with AA and GG homozygotes. Similarly, the risk assessed by BMI was 2.5 times lower in heterozygotes. Therefore, it can be speculated that the AG genotype may be a protective factor against obesity [46]. Further study by Maculewicz et al. [45] in a group of 139 physically active men (aged 19–29 years) showed no association between *IL-10* rs1518110, *IL-10* rs3024491, and *IL-10* receptor subunit beta, *IL-10RB* rs2834167 variants with obesity parameters defined as BMI, body fat percentage, and fat mass index. However, statistical significance was demonstrated in gene interactions for *IL-10RB* rs2834167 × *IL-10* rs1518110. The frequency of AG × AA genotypes was almost three times lower than that of AA × AA genotypes, and that of AG × AC was twice lower than for AA × A/C in the group with high tissue fat percentage [45].

#### 3.4.6. IL-15 Gene Polymorphisms

The results obtained in 108 Caucasian women (aged 20 to 45 years) showed that the change from A to G in the rs3136618 variant of IL-l5 receptor subunit α (*IL-15RA*) predisposes women to the De Lorenzo syndrome [29]. Individuals with Normal Weight Obese (NWO) syndrome have a substantial fat content of ≥30% despite having a BMI of ≤25 [51,52]. Furthermore, a trend for the prevalence of the *IL-15RA* rs3136617 A variant was found in NWO women. Finally, significant differences observed for the rs2296135-rs3136618 and rs3136617-rs2296135 haplotype distributions between overweight/obese and NWO women groups could support the hypothesis that genetic variability of the IL-15 receptor influences body fat composition [29].

#### 3.4.7. IL-18 Gene Polymorphisms

The *IL-18* rs1946518 (−607 C/A) and rs187238 (−137 G/C) promoter variants have been attributed to the higher transcription and protein production of IL-18 and have been associated with inflammatory diseases, cardiovascular risk, and insulin resistance. Genotype analyses of 680 Koreans (271 obese subjects, 170 female, aged 28.9–33.1 years) showed that rs1946518 A but not rs187238 variants may play a role in the development of obesity in women [31]. The association of the rs1946518 variant with an increased risk of obesity only in women was confirmed in a study of 560 Caucasians living in Western Siberia (220 obese subjects, 241 female, mean age of 59 years) [30].

Three IL-18 receptor accessory protein (*IL18-RAP*) gene polymorphisms were associated with body mass regulation. The study conducted in 1970 Caucasian individuals (aged 52.6 ± 18.7 years) from two different Spanish regions showed an association of rs7559479 G variant with a higher risk of obesity and body mass index, while the rs2293225 T and rs2272127 G variants were associated with a lower risk of obesity. The genetic variant rs7559479 is located within the 3′-UTR region of the *IL-18 RAP* gene, a target for microRNA 136. A functional assay demonstrated that the rs7559479 A variant reduces *IL-18RAP* mRNA levels and is associated with a lower risk of obesity [47].

### 3.5. Meta-Analysis

The search was conducted for all interleukin genes and described rs, but no consistent articles were found for the others. Genetic variants (rs) for all selected *IL* genes were examined. However, a meta-analysis could only be conducted for rs1800795, as the selected articles were the only ones that provided data on genotype frequencies for overweight/obese individuals and control groups, which is necessary for such an analysis. We focused on four studies that reported the association of *IL-6* rs1800795 for males only. Initially, we had 10 articles that included data for both women and men, but to enhance the specificity of our analysis, we selected only the four articles relevant to males. The chosen articles contained information or tables to infer the genotype frequency in overweight men and the control group.

**Figure 5:**
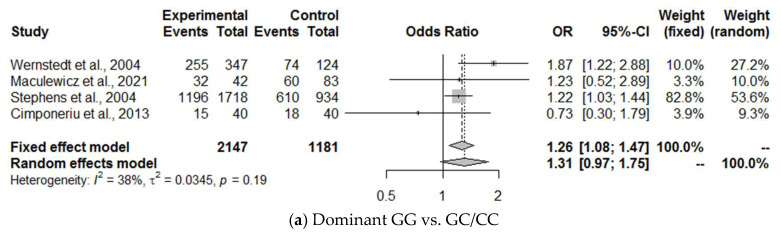

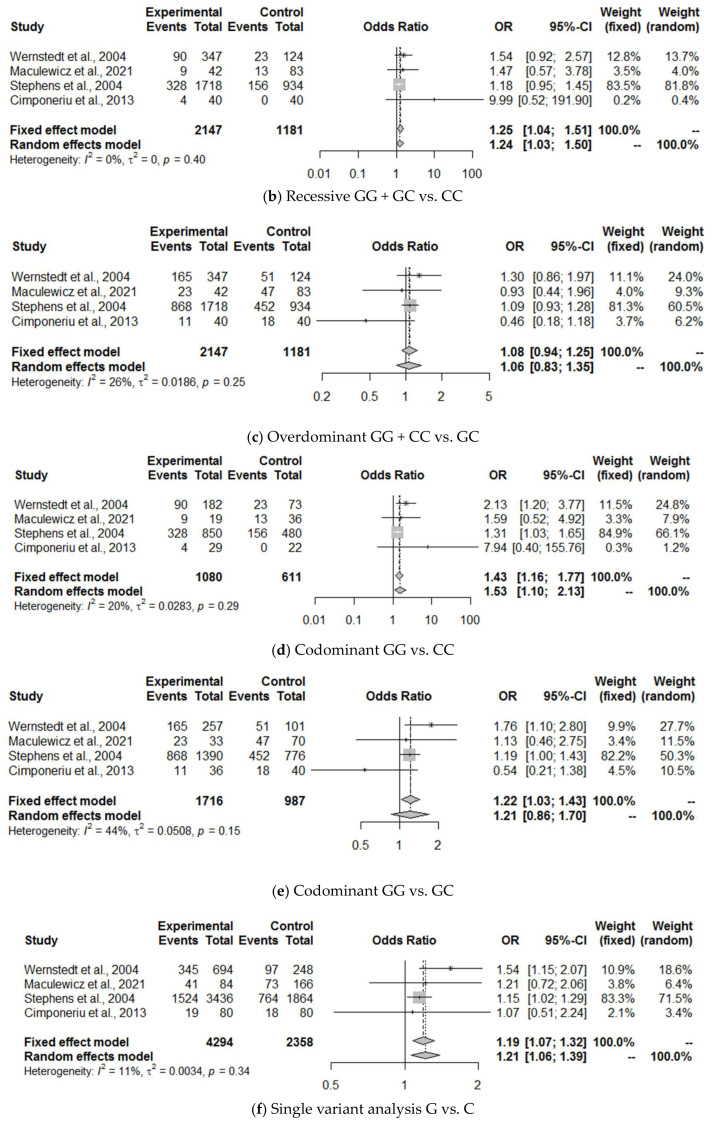
Forest plots of ORs for four articles on the association of *IL-6* rs1800795 with BMI in men. (**a**) GG vs. GC/CC, (**b**) GG + GC vs. CC, (**c**) GG + CC vs. GC, (**d**) GG vs. CC, (**e**) GG vs. GC, and (**f**) G vs. C. The grey area’s size indicates the weight of each study and the horizontal lines show the size of the 95% CI. References cited Wernstedt et al., 2004 [33], Maculewicz et al., 2021 [23], Stephens et al., 2004 [22], Cimponeriu et al., 2013 [27].

We calculated the OR for the dominant (GG vs. GC/CC), recessive (GG + GC vs. CC), overdominant (GG + CC vs. GC), and codominant (GG vs. CC and GG vs. GC) models and for the alleles (G vs. C) (Figure 5)

A fixed-effect model was chosen for dominant, recessive, overdominant, codominant (GG vs. CC), and alleles. Due to I^2^ > 40%, only the codominant (GG vs. GC) random-effect model was used. The pooled OR in the dominant model was 1.26 (95% CI 1.08 to 1.47). In Figure 5a, the dominant GG vs. GC/CC I^2^ was 38 and *p* = 0.019. Given that we selected a random-effect model for I^2^ > 40% and a fixed-effect model for I^2^ < 40%, this case falls just below our threshold for high heterogeneity. However, the fact that the *p*-value is <0.05 suggests that heterogeneity might be present.

In Figure 5b, the comparison of the recessive genotypes GG + GC vs. CC yielded an I^2^ value of 0% and a *p*-value of 0.4. This indicates the absence of heterogeneity and a non-significant *p*-value. Thus, we conclude that no statistical heterogeneity is present in this analysis. In Figure 5c, we can observe a low level of heterogeneity as I^2^ was only 26% and the *p*-value was >0.10. Thus, heterogeneity is low, and the non-significant *p*-value suggests that variability between studies is likely due to chance. In Figure 5d, I^2^ was low (20%) and the *p*-value > 0.10. This indicates minimal heterogeneity, suggesting that the studies are likely consistent in their effects. In Figure 5e, an I^2^ value of 44% indicates moderate heterogeneity. This suggests some variability in the effect sizes across the studies, though it is not high enough to be considered substantial. A *p*-value of 0.15 for the heterogeneity test was not statistically significant. This means that although there is some variability (I^2^ = 44%), it is not statistically significant, implying the differences in effect sizes are likely due to random sampling error rather than true differences between the studies. In Figure 5f, an I^2^ value of 11% indicates very low heterogeneity. A *p*-value of 0.34 for the heterogeneity test was not statistically significant. This indicates that the variability in effect sizes across the studies is likely attributable to random variation rather than genuine differences among the studies.

We showed that having the GC/CC genotype increased the likelihood of being overweight/obese, but the effect is limited. This finding, consistent with most published results, suggests that subjects with the GC/CC genotype are likelier to belong to a group with increased BMI. For the recessive model, a pooled OR of 1.25 (95% CI 1.04 to 1.51) means that subjects with the CC genotype were more likely to be overweight/obese. In the overdominant model, the pooled OR = 1.08 (95% CI 0.94 to 1.25) shows no practical association between the outcome and exposure, as the confidence interval (CI) includes 1. The codominant model showed that the CC and GC genotypes were associated with higher BMI values with a pooled OR = 1.43 (95% CI 1.16 to 1.77) and OR = 1.21 (95% CI 1.03 to 1.43), respectively. The single-variant analysis identified variant C as a risk variant associated with being in the higher BMI group. The pooled ORs for the dominant, overdominant, and codominant models indicated an increased likelihood of being overweight/obese, but the effect size was consistently below 1.5. However, in the recessive model, the effect size exceeded 1.5, suggesting a comparatively stronger association between the CC genotype and being overweight/obese. Therefore, based on these findings, it can be concluded that the maximum chance of being overweight/obese was less than 1.5 times higher for all models except the recessive model. The maximum chance of being overweight/obese was less than 1.5 times higher for all models except recessive. The effect is practically invisible in the recessive model.

### 3.6. Discussion

The current systematic review and meta-analysis included 136 studies investigating the association between IL genetic variants and overweight/obesity. Thirty were research articles evaluating overweight and obesity. The selected studies spanned a substantial 25-year timeframe (1998–2023).

The metabolic and immune systems are closely interrelated and functionally interdependent. Adipose tissue is crucial for energy storage and also acts as an endocrine organ by secreting bioactive substances that affect nearby tissues [53]. The composition of adipose tissue influences its secretory activity, which can be affected by genetic variation, potentially leading to systemic inflammation [54]. Obesity is associated with low-grade inflammation of white adipose tissue resulting from chronic activation of the innate immune system, which can subsequently lead to insulin resistance, impaired glucose tolerance, diabetes, and other metabolic disorders, including cardiovascular disease [55]. Cytokines and their immunomodulatory effects play an essential role in metabolic adaptation. The human immune system restores physiological homeostasis after infection or exercise [56]. Mounting evidence (also cited in the Review section) suggests that altered immune function resulting from genetic variability contributes to the pathogenesis of obesity. Therefore, IL polymorphisms appear to be associated with the metabolic processes occurring within adipose tissue.

One of the most important groups of inflammatory mediators involved in adipose tissue inflammation is the IL-1 family. Epidemiological studies have shown that *IL-1B* polymorphisms are associated with chronic diseases [42]. IL-1B is an early indicator of disturbed metabolic homeostasis and helps to restore balance by promoting acute adaptations. The highest concentration of IL-1 receptors is not found on immune cells but rather on insulin-producing β cells in rodents. This suggests that IL-1B is crucial in the immune system and metabolism interaction. During metabolic stress, it also inhibits mitochondrial metabolism [56]. Furthermore, IL-1B can activate the vagus nerve, which induces insulin secretion and regulates hypothalamic activity [57]. In experimental animals, studies have shown that *IL-1* receptor-deficient mice develop mature-onset obesity [58], and *IL-1Ra*-deficient mice become obesity-resistant [59].

In contrast to the roles played by most members of the IL-1 family, IL-18 was shown to inhibit inflammation and alleviate obesity and metabolic syndrome. Mice lacking IL-18 were shown to develop obesity. The administration of recombinant IL-18 reduced adiposity and caused mice to resist diet-induced metabolic dysfunction. Proposed mechanisms include energy expenditure, the activation of AMPK, and lipid oxidation in the muscle, and IL-18 is likely activated by NLRP1 inflammasome [60,61]. Anti-inflammatory IL-4 also protects against weight gain and metabolic syndrome. Michurina et al. (2024) deciphered that IL-4 activated glucose uptake and oxidation, lipid droplet fragmentation, fatty acid de-esterification, and thermogenesis in mature adipocytes [62].

The genetic variability of *IL-15* is of great importance in the context of body composition. It was found that IL-15 increases the lipolysis of adipocytes in vitro and decreases fat storage in cultured adipocytes [63]. Negative correlations between circulating IL-15 levels and adiposity have been demonstrated in humans [12]. Deletions of *IL-15* in humans and *Il-15* mice were associated with obesity, while gain-of-function *Il-15*-overexpressing mice were resistant to diet-induced obesity [64]. This protein is highly expressed in muscle [12] and acts as a muscular anabolic factor [65]. Pistillis et al. (2008) suggested the potential involvement of the IL-15 pathway in muscle and bone phenotypes and predictors of metabolic syndrome [66].

Specific effects on lipid, glucose, protein metabolism, and energy balance are best known for IL-6 [67]. Circulating IL-6 acts like a hormone, increasing energy availability by inducing lipolysis and fat oxidation, mobilizing fatty acids turnover [68], promoting hepatic glucose output [69], and improving insulin secretion and glucose tolerance [70]. When physiological concentrations of human recombinant IL-6 (rhIL-6) were administered to healthy young [71], elderly, and type 2 diabetic patients [72], it stimulated lipolysis and fat oxidation [71] and increased fatty acid turnover in elderly subjects with or without type 2 diabetes without affecting insulin sensitivity [72].

It was shown that brain-produced IL-6 was decreased in obese mice and rats, while intracerebroventricular IL-6 treatment suppressed body fat mass and prevented late-onset obesity in rats [73]. IL-6 microinjection reduced food intake and increased brown adipose tissue thermogenesis in male lean and obese rats, while an siRNA-mediated reduction in IL-6 led to increased weight gain and adiposity, which affects hypothalamic function [13]. Furthermore, blocking IL-6 activity in humans with receptor antagonist *tocilizumab* increases visceral fat mass and fatty acid storage [74]. In infection or acute exercise, IL-6 has been described to suppress appetite and food intake and delay gastric emptying [56,75]. In contrast, the results of Bornath et al. (2023) did not suggest any exercise-induced anorexigenic effects of IL-6 [76]. Notably, increased serum levels of the cytokine IL-6 accompany obesity [77,78,79] and decrease with weight loss [55,79,80].

Determining the variability of *IL-6* and studying functional variants provide insight into its role in normal and pathological metabolism [4,81]. Different variants in the *IL-6* gene have been attempted to be associated with various metabolic disorders and physiological phenotypes. Kubaszek et al. reported that the *IL-6* rs1800795 C promoter variant slightly decreased energy expenditure and restrained transcription in different cell systems, leading to increased adiposity [82]. An analysis of this variant’s variability needs to be clarified.

Our meta-analysis concluded that the *IL-6* rs1800795 C variant may confer a population-attributable risk of overweight/obesity. The low OR and the wide CI calculated in our statistical analysis suggest that the environment may reduce, eliminate, or enhance the functional effects of *IL-6* gene variability. We initially included ten articles in our meta-analysis, excluded studies involving children and adolescents, and finally focused on only males. This approach both restricted the research to fewer subjects and enabled us to conduct the meta-analysis on a potentially homogeneous group. Although ethnically and sexually homogeneous groups were selected, the statistical analysis appears to be influenced by health status, age, and lifestyle differences.

The *IL-6* rs1800795 GC/CC genotypes were significantly more common in obese men in almost all publications analyzed. Stephens et al. (2004) even described a linear association between genotype and BMI. When the subjects with type 2 diabetes were stratified by median BMI, 62% of CC subjects were in the higher group compared to 38% in the lower group. This stratification was not achieved when healthy men were similarly stratified [22]. It seems that the variant may play other roles as a risk factor for obesity and as a factor already involved in disease progression.

Our study indicates that including those who are obese as a result of their disease and still healthy but overweight people in the same analysis can be misleading. Similarly, combining different groups of patients based on their obesity alone can also be misleading. Depending on the stage of the disease, we may receive inconsistent results from multiple analyses. Biological markers related to the cardiovascular system increasingly deteriorate in the group of obese people with metabolic disorders, indicating the development of an obesogenic environment. This obesogenic environment may be necessary for the complete genetic manifestation and, on the other hand, may redistribute the genotype in the population due to reduced life expectancy. Stephens et al. (2004) indicated a lower frequency of the *IL-6* rs1800795 C variant in the group of the most obese diabetic patients compared to controls. The unexpected genotype distribution was not caused by genotype protection but rather was likely due to the comorbidity of insulin resistance with the cardiovascular disease in this highest-risk group and the earlier death of CC carriers [22]. The effect of the rs1800795 variant may also be modulated by the immune response to infection, as Cimponeriu et al. (2013) suggested. The authors examined the genotypes in lean and obese subjects infected with the Torque teno virus (TTV) in the Romanian population. They showed that only the CC genotype was significantly more common in obese individuals. In contrast to most studies, the CG genotype was not a risk factor in this population. TTV was found more frequently in the subgroup of obese women than in controls, which could influence the genotype distribution [27]. Notably, the *IL-6* promoter region, in which the rs1800795 polymorphism was mapped, contains essential cis-acting regulatory elements responsible for virus-induced gene transcription [83]. It could be speculated that when a solid environmental factor promotes obesity, weak genetic influences become insignificant. Supporting this, Maculewicz et al. (2021) showed that the rs1800795 variant had no effect in a very homogeneous group of young male military professionals engaged in high levels of physical activity and a diet according to the recommendation for military professionals [23]. These results highlight that environmental factors can effectively counteract genetic influences. If a healthy lifestyle is maintained, the effects of the rs1800795 variant may be negligible.

Wernstedt et al. (2004) reached similar conclusions, finding that the difference of 1.5 kg/m^2^ in BMI that may result from CC homozygosity is substantial but not critical for severe obesity [33]. However, weight gain may contribute to a vicious circle of negative processes. Previous studies on the genetic basis of obesity indicate that it is tough to assess the impact of a single genomic variant on such a multifaceted phenomenon in which, in addition to environmental factors and biological predisposition, intertwined psychological, social, cultural, and economic conditions may play an important, if not decisive, role. Moreover, obesity can be both a cause and a consequence of metabolic disorders, making it difficult to assess as a stand-alone trait without taking into account chronic comorbidities. Thus, conflicting findings on the effect of genetic variation may indicate a relationship between the variant and disease susceptibility rather than obesity per se.

This is the first systematic review and meta-analysis to evaluate the role of IL gene polymorphisms in overweight/obesity. This study helps better understand the relationship between inflammatory components and susceptibility to obesity. In a complex condition such as obesity, multiple factors with diverse mechanisms play roles.

Identifying specific genetic variants, particularly the *IL-6* rs1800795 polymorphism, as modestly associated with obesity risk suggests a need for more personalized approaches in obesity management. Clinicians may consider these genetic insights when designing intervention programs. For example, individuals with a greater genetic susceptibility might achieve better results from customized lifestyle modification plans that prioritize dietary adjustments and exercise routines. Additionally, the implementation of genetic testing in clinical settings could improve the identification of individuals at elevated risk for obesity, facilitating early interventions and more focused counseling efforts.

However, our study only focused on a single genetic risk factor, and we did not account for numerous other contributing cultural and environmental factors. Further studies of the obesity phenomenon should use large-scale methods, both to study large groups of individuals and to simultaneously analyze the results of variation at multiple omic levels (primarily the metabolome, proteome, and transcriptome) in the context of the total phenotypic picture and the genomic variants possessed by specific individuals. Next, one notable limitation of the meta-analysis is the inclusion of only four studies focusing specifically on the *IL-6* rs1800795 polymorphism. This limited sample size significantly reduces the statistical power of the analysis and may affect the reliability of the pooled odds ratios. The small number of studies can also introduce variability and bias, as the results may not fully represent the broader population or the complexity of the interaction between IL-6 variants and obesity.

The review processes employed in this study, while systematic and comprehensive, are subject to several limitations that should be acknowledged. Although the Cochrane Collaboration tool was utilized to assess the risk of bias in the included studies, the subjective nature of this assessment can lead to inconsistencies. Different reviewers may interpret the criteria for bias differently, which can affect the overall assessment of the studies. This subjectivity might overlook specific biases related to study design or reporting. The reliance on published studies may introduce publication bias, as studies with significant or positive results are more likely to be published than those with null or negative findings. This bias could skew the meta-analysis results, as it may not fully reflect the true relationship between interleukin gene polymorphisms and obesity. The review was constrained to studies published from 1998 to 2023. While this temporal framework is pertinent, it may overlook significant earlier research and studies from 2024, which could offer essential context or foundational insights. Additionally, the inclusion criteria for selecting studies could have excluded relevant research due to strict definitions, limiting the breadth of literature considered.

## 4. Conclusions

Unlike many previous reviews, which might concentrate on a single cytokine gene or a limited set of genetic factors, this review evaluated multiple subsets of cytokine genes, namely interleukin genes. Thirty articles were included in the systematic review, and four were included in the meta-analysis. Based on those 30 articles, the current review indicates that variations in interleukin genes can affect an individual’s vulnerability to overweight or obesity, providing insights into the genetic factors influencing these conditions. The results concerning the *IL-4* rs1800795 polymorphism indicate a potential, albeit modest, association with obesity phenotypes. Individuals possessing particular genotypes at this locus may exhibit an elevated probability of being categorized as overweight or obese relative to those with alternative genotypes. Nevertheless, the magnitude of this association remains limited, suggesting that other genetic, environmental, and lifestyle factors may exert a more pronounced influence on weight regulation. Therefore, while the *IL-4* rs1800795 variant may contribute to the risk of obesity, it represents only one facet of a multifactorial interplay affecting this condition. Future research should focus on more extensive and diverse cohort studies to validate the *IL-4* polymorphism’s association with obesity across different populations. Longitudinal studies are crucial for assessing how the variant affects weight and fat gain over time. Additionally, haplotype analysis in the *IL-4* gene and genotypic interactions will help clarify the impact on obesity risk.

## Figures and Tables

**Table 1:** A list of variants included in the review section (population, cohort).

Interleukin Gene	Polymorphism (rs)	Author	Population	Cohort (Sex, Sample Size)
*IL-6*	1800795, 1800796	Wernstedt et al., 2004 [33]	Caucasian	355 males, 204 females
*IL-6*	1800795	Gupta et al., 2010 [20]	Caucasian	370 females
*IL-6*	1800795, 1800796	Mărginean et al., 2016 [34]	Caucasian	309 females and 309 newborns of unknown sex
*IL-6*	1800795, 1800796	Oana et al., 2014 [21]	Caucasian	114 males, 98 females
*IL-6*	1800795	Panoulas et al., 2009 [35]	Caucasian	270 males, 535 females
*IL-6*	1800795	Stephens et al., 2004 [22]	Caucasian	2652 males
*IL-6*	1800797, 1800796, 1800795	Boeta-Lopez et al., 2018 [36]	Mexican Americans	146 males, 291 females
*IL-6*	1800795, 1800796, 13306435	Maculewicz et al., 2021 [23]	Caucasian	125 males
*IL-6*	1800795	Goyenechea et al., 2007 [24]	Caucasian	54 males, 52 females
*IL-6*	1800795	Klipstein-Grobusch et al., 2006 [25]	Caucasian	334 males, 334 females
*IL-6*	1800795, 1800797, 1800796	Cozen et al., 2006 [26]	non-Latino White (White), African American, Latino White (Latino), Asian/other	218 males, 170 females
*IL-6*	8192284	Song et al., 2007 [37]	Asian	321 males, 37 females
*IL-6*	1800795	Berthier et al., 2003 [38]	French-Canadian	270 males
*IL-6*	1800795	Cimponeriu et al., 2013 [27]	Caucasian	80 males, 220 females
*IL-6*	2228145, 2229238, 4845623	Wolford et al., 2003 [39]	Pima Indian	1813 males, 1920 females
*IL-6*	1800795, 1800797, 1800796	Hamid et al., 2005 [28]	Caucasian	3919 males, 3640 females
*IL-1*	1143634	Strandberget al., 2006 [40]	Caucasian	1068 males
*IL-1*	1143634	Strandberg et al., 2008 [41]	Caucasian	3014 males
*IL-1*	1143634	Lee et al., 2008 [5]	Asian	181 females
*IL-1*	1143634	Manica-Cattani et al., 2010 [42]	Caucasian	880 participants of an unknown number of males and females
*IL-1*	16944, 1143623, 4848306, 1143633, 17561, 1143634	Wilkins et al., 2017 [32]	288 White and 4 Black	292 males
*IL-1*	1800587,1143634, 2234677	Maculewicz et al., 2022 [43]	Caucasian	101 males
*IL-10*	1518111, 1878672, 3024496, 3024498, 3024505	Ha et al., 2008 [44]	Asian	446 males, 421 females
*IL-10*	1800896, 1800871, 1800872	Maculewicz et al., 2022 [45]	Caucasian	139 males
*IL-10*	1800896, 1800871, 1800872	Scarpelli et al., 2006 [6]	Caucasian	759 males, 923 females
*IL-10*	1518111, 1878672, 3024496, 3024498, 3024505	Maculewicz et al., 2022 [46]	Caucasian	131 males
*IL-15*	3136617, 3136618, 2296135	Di Renzo et al., 2009 [29]	Caucasian	108 females
*IL-18*	187238	Ponasenko et al., 2022 [30]	Caucasian	319 males, 241 females
*IL-18*	1946518, 187238	Kim et al., 2012 [31]	Asian	101 males and 170 females; 409 controls of unknown sex
*IL-18*	7559479, 2293225, 2272127	Martínez-Barquero et al., 2017 [47]	Caucasian	966 males, 1006 females

**Table 2:** A list of variants included in the review section (age, body composition, and additional information).

Interleukin Gene	Polymorphism (rs)	Author	Age	Body Composition Assessment	Additional Information
*IL-6*	1800795, 1800796	Wernstedt et al., 2004 [33]	56.8 ± 0.31	normal weight, overweight	healthy, and with hypertension, diabetes not excluded
*IL-6*	1800795	Gupta et al., 2010 [20]	obese: 29.12 ± 6.51, non-obese: 28.04 ± 5.86	non-obese, obese	obese group had significantly higher values for blood glucose, CRP-HOMA-IR
*IL-6*	1800795, 1800796	Mărginean et al., 2016 [34]	29.20 ± 5.44	normal weight, overweight, obesity	0.60% diabetics, 4.50% gestational arterial hypertension
*IL-6*	1800795, 1800796	Oana et al., 2014 [21]	obese: 9.61 ± 3.9, non-obese: 10.67 ± 4.54	normal weight, obesity	control patients with normal nutritional status, and obese patients
*IL-6*	1800795	Panoulas et al., 2009 [35]	RA patients: 61.3 ± 12.1, controls: 50.1 ± 15.7	overweight, obesity	cardiovascular diseases, RA, smoking
*IL-6*	1800795	Stephens et al., 2004 [22]	obese: 64.4 (10.4), non-obese: 69.2 (10.8)	normal weight, overweight, obesity	metabolic syndrome including elevated systolic and diastolic blood pressure, triglycerides, total cholesterol: HDL-cholesterol ratio, CRP, and lower HDL
*IL-6*	1800797, 1800796, 1800795	Boeta-Lopez et al., 2018 [36]	17 ± 8.26	normal weight, overweight, obesity	HDL, HOMA-IR, LDL
*IL-6*	1800795, 1800796, 13306435	Maculewicz et al., 2021 [23]	CONBMI 21.87 ± 1.57, OVERBMI 21.98 ± 1.79, CONFat 21.92 ± 1.79, OVERFat 21.84 ± 1.87	normal weight, overweight, obesity	physical activity
*IL-6*	1800795	Goyenechea et al., 2007 [24]	median (IQR): 34 (25–43)	obesity	metabolic disorders, such as hypertension, atherogenic dyslipidemia, and insulin resistance evaluated by the homeostasis model assessment of insulin resistance index
*IL-6*	1800795	Klipstein- Grobusch et al., 2006 [25]	obese: 53.9 ± 7.3, non-obese: 53.8 ± 7.3	normal weight, obesity	high blood pressure, smoking status, prevalence of T2DM, and myocardial infarction
*IL-6*	1800795, 1800797, 1800796	Cozen et al., 2006 [26]	median age = 61	normal weight, overweight, obesity	risk of multiple myeloma and plasmacytoma
*IL-6*	8192284	Song et al., 2007 [37]	46.1 ± 11.5	normal weight, overweight, obesity	dietary interactions
*IL-6*	1800795	Berthier et al., 2003 [38]	GG genotype: 41.83 ± 7.39, GC genotype: 42.64 ± 7.89, CC genotype: 43.64 ± 8.77	obesity	body fat distribution (waist circumference, subcutaneous and visceral adipose tissue), and metabolic responses (plasma glucose and insulin levels during an oral glucose tolerance test)
*IL-6*	1800795	Cimponeriu et al., 2013 [27]	obese: 43.13 ± 12.20, non-obese: 44.21 ± 11.95	normal weight, overweight, obesity	viral infections (TTV), hypertensive status (resting blood pressure exceeding 130/85 mm Hg or subject under antihypertensive treatment), smoking(more than five cigarettes per day for at least one year), and drinking (at least five units of alcohol per day for at least one year)
*IL-6*	2228145, 2229238, 4845623	Wolford et al., 2003 [39]	NA	normal weight, overweight, obesity	T2DM
*IL-6*	1800795, 1800797, 1800796	Hamid et al., 2005 [28]	57 ± 11	obesity	Inter99 cohort without metabolic syndrome, type 2 diabetic patients
*IL-1*	1143634	Strandberg et al., 2006 [40]	18.9 ± 0.6	obesity	Gothenburg Osteoporosis and Obesity Determinants (GOOD) study group
*IL-1*	1143634	Strandberg et al., 2008 [41]	69–81	overweight	the Swedish part of the Osteoporotic Fractures in Men (MrOS) multicenter study
*IL-1*	1143634	Lee et al., 2008 [5]	18–62	normal weight, overweight, and obesity	divided into three BMI groups
*IL-1*	1143634	Manica-Cattani et al., 2010 [42]	18–92	normal weight, overweight, and obesity	divided into three BMI groups
*IL-1*	16944, 1143623, 4848306, 1143633, 17561, 1143634	Wilkins et al., 2017 [32]	29–64	obesity	the study group from the Veterans Affairs Dental Longitudinal Study (DLS), free of chronic medical conditions at the start of the study
*IL-1*	1800587,1143634, 2234677	Maculewicz et al., 2022 [43]	OVERBMI 21.8 ± 1.64, CONBMI 21.9 ± 1.61, OVERFat 21.4 ± 1.6, CONFat 21.9 ± 1.6	normal weight, overweight	two control and two study groups based on two criteria: BMI and fat percentage, healthy, physical activity
*IL-10*	1518111, 1878672, 3024496, 3024498, 3024505	Ha et al., 2008 [44]	43.75 years ±12.73	normal weight, moderately obese, and obesity	divided into three BMI groups
*IL-10*	1800896, 1800871, 1800872	Maculewicz et al., 2022 [45]	CONBMI 22.02 ± 1.86, OVERBMI 22.63 ± 2.36, CONFMI 22.10 ± 1.92, OVERFMI 24.10 ± 2.95, CONFat 22.13 ± 1.89, OVERFat 2.83 ± 2.78	normal weight, overweight	three control and three study groups based on two criteria: BMI and fat percentage, healthy
*IL-10*	1800896, 1800871, 1800872	Scarpelli et al., 2006 [6]	47 ± 14 nondiabetics, 61 ± 11 diabetics	normal weight, overweight, and obesity	non-diabetic control, patients with T2DM
*IL-10*	1518111, 1878672, 3024496, 3024498, 3024505	Maculewicz et al., 2022 [46]	OVERBMI 22.6 ± 2.5, CONBMI 22.3 ± 2.1 OVERFat 22.30 ± 2.3 CONFat 22.4 ± 2.2	normal weight, overweight	two control and two study groups based on two criteria: BMI and fat percentage, healthy, physical activity
*IL-15*	3136617, 3136618, 2296135	Di Renzo et al., 2009 [29]	20–45	non-obese, normal weight obese, preobese-obese	three groups based on body composition (non-obese, normal weight obese, preobese-obese), healthy, physical activity
*IL-18*	187238	Ponasenko et al., 2022 [30]	≤60 and >60	non-obese, obese	two groups based on body composition (non-obese, obese), healthy
*IL-18*	1946518, 187238	Kim et al., 2012 [31]	moderately overweight 28.9 ± 11.2, severely overweight 31.3 ± 10.54, obese 33.1 ± 12.9	moderately overweight, severely overweight, and obese	three groups based on BMI, healthy
*IL-18*	7559479, 2293225, 2272127	Martínez-Barquero et al., 2017 [47]	VALCAR 46.4 ± 14.9, Hortega 54.4 ± 19.3	non-obese, obese	VALCAR and Hortega groups (at cardiovascular risk)

CONBMI—control BMI, CONFat—control fat, CRP-C—reactive protein, HDL—high-density lipoprotein cholesterol, HOMA-IR—homeostasis model assessment of insulin resistance, LDL—low-density lipoprotein cholesterol, NA—non-applicable, OVERBMI—over BMI, OVERFat—over fat, RA—rheumatoid arthritis, T2DM—type 2 diabetes, TTV—Torque teno virus.

**Table 3:** Summary of the risk of bias for each study included in the review based on [18].

Publication Reference	Random Sequence Generation	Allocation Concealment	Blinding of Participants and Personnel	Blinding of Outcome Assessment	Incomplete Outcome Data	Selective Reporting	Other Bias
e.g., [reference number]	Criteria for a judgement of ‘Low risk’ of bias. e.g., low	Criteria for a judgement of ‘Low risk’ of bias. e.g., low	Criteria for a judgement of ‘Low risk’ of bias. e.g., low	Criteria for a judgement of ‘Low risk’ of bias. e.g., low	Criteria for a judgement of ‘Low risk’ of bias. e.g., low	Criteria for a judgement of ‘Low risk’ of bias. e.g., low	Criteria for a judgement of ‘Low risk’ of bias. e.g., low
	Criteria for the judgement of ‘High risk’ of bias. e.g., low	Criteria for the judgement of ‘High risk’ of bias. e.g., low	Criteria for the judgement of ‘High risk’ of bias. e.g., low	Criteria for the judgement of ‘High risk’ of bias. e.g., low	Criteria for the judgement of ‘High risk’ of bias. e.g., low	Criteria for the judgement of ‘High risk’ of bias. e.g., low	Criteria for the judgement of ‘High risk’ of bias. e.g., low
	Criteria for the judgement of ‘High risk’ of bias. e.g., low	Criteria for the judgement of ‘High risk’ of bias. e.g., low	Criteria for the judgement of ‘High risk’ of bias. e.g., low	Criteria for the judgement of ‘High risk’ of bias. e.g., low	Criteria for the judgement of ‘High risk’ of bias. e.g., low	Criteria for the judgement of ‘High risk’ of bias. e.g., low	Criteria for the judgement of ‘High risk’ of bias. e.g., low

## Data Availability

All data supporting the findings of this study are available within the manuscript. The datasets generated and analyzed during the current study are not publicly available but are available upon reasonable request from the corresponding author, who was an organizer of the study.
